# Collective behavior quantification on human odor effects against female *Aedes aegypti* mosquitoes—Open source development

**DOI:** 10.1371/journal.pone.0171555

**Published:** 2017-02-02

**Authors:** Abdul Halim Poh, Mahmoud Moghavvemi, Cherng Shii Leong, Yee Ling Lau, Alireza Safdari Ghandari, Alexlee Apau, Faisal Rafiq Mahamd Adikan

**Affiliations:** 1 Department of Electrical Engineering, Faculty of Engineering, University of Malaya, Kuala Lumpur, Malaysia; 2 Centre of Research in Applied Electronics, Faculty of Engineering, University of Malaya, Kuala Lumpur, Malaysia; 3 University of Science and Culture, Tehran, Iran; 4 Department of Parasitology, Faculty of Medicine, University of Malaya, Kuala Lumpur, Malaysia; National Taiwan Ocean University, TAIWAN

## Abstract

Classifying and quantifying mosquito activity includes a plethora of categories, ranging from measuring flight speeds, repellency, feeding rates, and specific behaviors such as home entry, swooping and resting, among others. Entomologists have been progressing more toward using machine vision for efficiency for this endeavor. Digital methods have been used to study the behavior of insects in labs, for instance via three-dimensional tracking with specialized cameras to observe the reaction of mosquitoes towards human odor, heat and CO_2_, although virtually none was reported for several important fields, such as repellency studies which have a significant need for a proper response quantification. However, tracking mosquitoes individually is a challenge and only limited number of specimens can be studied. Although tracking large numbers of individual insects is hailed as one of the characteristics of an ideal automated image-based tracking system especially in 3D, it also is a costly method, often requiring specialized hardware and limited access to the algorithms used for mapping the specimens. The method proposed contributes towards (a) unlimited open source use, (b) a low-cost setup, (c) complete guide for any entomologist to adapt in terms of hardware and software, (d) simple to use, and (e) a lightweight data output for collective behavior analysis of mosquitoes. The setup is demonstrated by testing a simple response of mosquitoes in the presence of human odor versus control, one session with continuous human presence as a stimuli and the other with periodic presence. A group of female *Aedes aegypti* (Linnaeus) mosquitoes are released into a white-background chamber with a transparent acrylic panel on one side. The video feed of the mosquitoes are processed using filtered contours in a threshold-adjustable video. The mosquitoes in the chamber are mapped on the raster where the coordinates of each mosquito are recorded with the corresponding timestamp. The average distance of the blobs within the frames against time forms a spectra where behavioral patterns can be observed directly, whether any collective effect is observed. With this method, 3D tracking will not be required and a more straightforward data output can be obtained.

## I. Introduction

Automated mosquito behavioral studies mainly consists of the use of several devices such as digital video cameras, paired with computers for logging data and an observatory of various dimensions to contain the mosquitoes. However, arbitrary platforms of observation poses a challenge for researchers to refine upon since variables and methodology differ from study to study. Studies on automated documentation of mosquito activity include quantifying the flight speeds in 2D [1] and 3D [2, 3], heuristic classification of activities such as swooping, visiting, bouncing and resting [4], landing rates [5] and house-entry behaviour [6], among others. Previously done manually, entomologists are progressing toward digitized methods on quantifying mosquito behaviour, which has obvious advantages including capability of analysing large amounts of tracking data and draw more accurate conclusions using advanced statistical analyses [7]. However, due to the complexity and limitations of these methods, many attributed to the limitations of computer vision and access to the software used, much of the studies which requires these fundamental issues to be resolved remains a challenge for entomologists. For instance, based on reviews made on several selected studies, there seems to be unanimous conclusions made on the inefficacy of electronics-based repellents for mosquitoes, where either the use of radio frequencies (RF) or ultrasonic methods in the reviews are not specified. However, whether man-made electromagnetic (EM) signals throughout the spectrum have any effect on mosquitoes has yet been established, and reports on research in this discipline using automated quantification setups on this area has been virtually non-existent [8]. This is due to the scarcity of systematic studies and the technical difficulties surrounding the setup, where only a few selected frequencies between 125–74,600 Hz was tested where details of electronic components and circuitry was not mentioned [8]. Recent mosquito repellent studies also still use manual counting methods, as prescribed by the World Health Organization (WHO) where the repellant/insecticidal capability of a certain medium is measured by counting the number of mosquitoes knocked down after regular intervals, as employed by recent studies as well [9]. Other recent studies also adopted arm-in-cage assays, where manual counting of landed mosquitoes are performed [10, 11]. The indexing methods on mosquito activity are defined as spatial activity index (SAI), or feeding/inhibition rates [12] and landing frequency. Although there were safety concerns on such methods, it has been proven to be a non-issue [13]. Manual counting of *Ae*. *aegypti* mosquito eggs on a paper filter are also still used in a recent study on investigating the deterrent effect of essential oils from torch ginger (*Etlingera elatior*, Zingiberaceae) against oviposition [14].

The main issue from these counting methods, however, is the counting procedure itself which is left to ambiguity of manual observation. This was probably due to the reluctance of movement ecologists to adopt graph theory to quantify the behaviour of insects [15]. Several automated insect behavior quantification methods have been reported with varying degrees of success, including an optical counting method reported by Hoffmann *et*. *al* in 2010 where mosquitoes passing a line are recorded and counted. [16]. A recent mosquito-zapper was developed by using machine vision coupled with laser beams to compute the wingbeat, which is then mapped and shot with the laser beam [17]. The latest digital methods of analyzing mosquito behavior was also reported using 3D tracking and analyzing the behavior for response against human odor and heat [2], thermal effects with CO_2_ [18], visualizing home entry [6] and insecticide impact on bed nets [4, 19]. Reconstruction of 3D trajectories of the flights have also been attempted in more recent studies, highlighting improved models [20, 21]. Stereoscopic video analysis has been used to study swarm behavior in fruit flies [22] and male *Anopheles gambiae* mosquitoes with emphasis on probable mating habits [23], while 3D tracking on the latter have also been demonstrated using probabilistic framework, although the method was carried out purely addressing the flight path, with some issues with accuracy due to occlusion [24]. A similar study was also conducted using pan-tilt cameras for flight tracking of bees [25]. A Matlab-based, open-source development was also reported, for analyzing and tracking bees using tag designs [26]. However, in contrast to bees due to the complexity of 3D analysis and smaller mosquito sizes, automated 3D tracking of multiple individual mosquitoes remains a nontrivial issue due to the mercurial nature of the specimens in digital forms, although challenges in 3D tracking has not been to confined to mosquitoes only [7, 22, 24]. A method to analyze *Drosophila* using machine vision by computing the contours in an image using Gaussian mixture model at a very close range was also used [27], although bearing similarity in this paper, the complexity of the analysis is not required in our study. From these previous studies, a few qualities need to be summarized for an automated data collection on mosquito activity, which includes the need for a collective behavior analysis addressing a mosquito swarm, open-source software, low-cost and does not require 3D tracking since it leads to data complexity and confusion. This is further supported by a study which reports the adequacy of a 2D analysis for mosquito flight activity at 90% accuracy [1]. Therefore, this paper reports a simpler method on analyzing collective behavior of *Ae*. *aegypti* (Linnaeus) mosquitoes, using machine vision by using low-cost USB-connected cameras, and utilizing image processing techniques, available in an open-source development platform OpenCV^TM^ 3.0 coded in C++ environment with Microsoft Visual Studio^TM^ 2013. To demonstrate the potential and advantages of this method, an applied research similar to to previous studies [2, 5] is carried out by analyzing mosquito response against human odor, which fundamentally requires only an observation chamber with mosquitoes, a USB-connected camera paired with a computer, and a human as stimuli.

To meet the specific requirements in this work, some aspects will be considered for the quantification to occur systematically, which includes the chamber design, digital mosquito behavior quantification method using low cost USB-connected web camera, the software development for the image processing, data management and finally the analytics. The issues faced in this particular work is mentioned for future improvement towards low-cost automated quantification on mosquito swarm behavior, which can be in the form of improving the source code and the hardware setup.

## II. Materials & methods

### A. Chamber design and experimental setup

**Fig 1:**
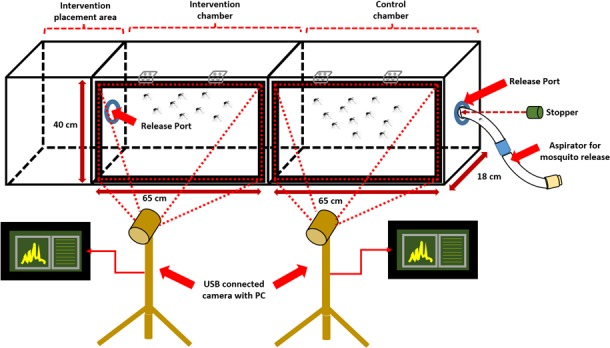
Chamber for Mosquito Behavior Analysis.

Different mosquito chamber designs for different applications as reported in various studies have been studied for a comparative guide on the setup used in this paper [12, 28, 29]. The chamber for this purpose is a wooden box (sized 176 x 47 x 18 cm) with an acrylic panel to allow view of the mosquitoes’ motion. However, in this test, we observe only two-dimensional components, namely the *x-* and *y-* components for simplification, also supported by the studies on mosquito flight speeds in 2D [1]. This is done by limiting the length or the third dimension of the chamber to 18 cm in contrast to the width of 65cm and height of 40 cm, which renders the *z*-axis movements of mosquitoes as negligible in effect. The chamber consists of two parts which is the test region and a control region. Both regions including the release ports are completely isolated with general purpose silicone sealant and elastomeric nitrile foamed rubber (Superlon^TM^) for added security. Fig 1 shows the complete setup for the test:

Two standard issue USB-connected cameras are used for the intervention and control regions, where both these regions will be referred to according to the aforementioned designation throughout this paper. The cameras are placed in front of the chamber and adjusted accordingly to get the best resolution while covering a considerable region for any mosquito activity to be captured. The background colour of choice is white, to provide best contrasting effects between the mosquitoes and the background as also preferred by in-lab tests [15], which is required for the background subtraction algorithms.

This design has several reasons, primarily designed for collective behaviour aggregation of the mosquitoes in 2D, and also to reduce the load for the algorithms where the area of interest will be limited to the chamber only. The dimensions are deemed suitable since human presence-detection tendencies are exhibited when subjects are within 30 cm as demonstrated with *Anopheles gambiae sensu stricto* Mosquitoes [2], and 18-cm width is approximately proportionate with 2D analysis used in previous studies, which was 10 cm [1]. Also this is done for a low-cost camera resolution to be effective where too large a chamber will require a greater distance from the chamber, which will render the camera resolution unacceptable.

### B. Hardware setup & image processing algorithm

**Fig 2:**
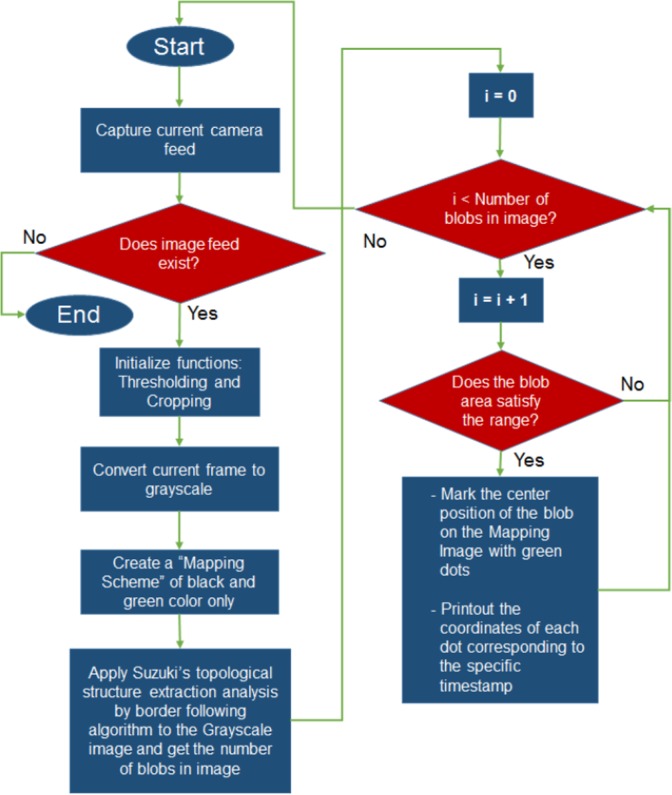
Image Processing Algorithm Flowchart.

Two standard USB-connected web cameras are used in this study (Sirius USB2.0 and Logitech S7500). The cameras are mounted using custom-made brackets for use on a tripod facing the chamber as shown in Fig 1. For detecting the mosquitoes from the background in the image, a custom made Graphical User Interface (GUI) was developed. Suzuki’s method of topological structure extraction analysis by border following [30] algorithm available was used as a function adapted in an open-source image processing software, OpenCV^TM^ [31] coded in C++ environment with Microsoft Visual Studio^TM^ 2013 (**See S1 Source Code and Files**). The method allows blobs to be extracted from a grayscale image, and a blob which satisfies certain characteristics will be construed as a mosquito within the raster array. The algorithm used is explained in Fig 2.

Using Suzuki’s method [30], considering a binary image after converting a current digital image obtained from the camera into grayscale, the threshold of the grayscale image is adjusted until the mosquitoes are visible within the binary form. The uppermost to lowermost rows and leftmost to rightmost columns compose a frame. 1-pixels and 0-pixels are designated for the pixels with 1 and 0 densities respectively. Several conditions were imposed for the algorithm. First, the surrounding frame pixels are all assumed as 0-pixels. Any value can be assigned to each pixel during the processing where the coordinates begin at the leftmost column and topmost row pixel, denoted as (i,j). The picture with density f_ij_ at pixel (i,j) is indicated as F = {f_ij_}. Connected 1- and 0-pixels are construed as the 1- and 0-component *S* where if a 1-component contains a frame, it is regarded as a hole and otherwise, a background. 0-pixels are regarded as 8- (4-) connected (as per Moore Neighborhood cellular automation) as 1-pixels are 4- (8-) connected (as per von Neumann Neighborhood) to avoid topographical discrepancy.

The borders are further defined as follows: With 4- (8-) connected case, a 1-pixel (i,j) having 0-pixels (p,q) *S*_2_ in its 8- (4-) neighborhood is defined as a border point, or simply as the point between a 1-component S_1_ and 0-component *S*_2_. Then, if a pixel belonging to *S*_2_ is in *S*_1_, *S*_2_ is defined as surrounding S_1_ and vice versa. If a border exists between them, *S*_2_ is defined as directly surrounding *S*_1_. An outer border is further construed as a set of border points which is surrounded either by 1- or 0-components. In the case of multiple hole/background within a border, a parent border is the hole border between *S*_2_ and the 1-component enveloping *S*_2_, if *S*_2_ is a hole, whereas if *S*_2_ is the background, is the frame of the picture. In addition, for two borders B_0_ and B_n_, B_0_ is said to be enveloped by B_n_ if a sequence of border B_0_, B_1_, … B_n_ exists.

Then the border following algorithm for topological analysis is defined in the following, where an input binary frame is interrupted when a pixel (i,j) satisfies the conditions for the border following, whether it is an outer border or a hole border. A formed border is further numbered and kept in the computer memory. The parent border is determined by keeping the sequence of the newly found borders, where it will be matched with the type. Following a classical border following scheme [32], after the marking entire borders, the raster scan is continued until the last pixel in the right corner of the array is read. This marks the end of the algorithm.

**Fig 3:**
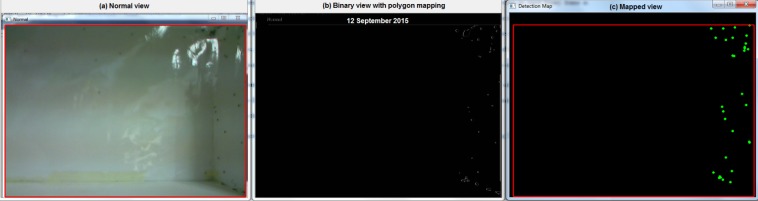
GUI demonstration on the mosquitoes. (a) The leftmost is the original image, (b) indicates the Douglas-Peucker algorithm-applied polygons constructing the blobs around the detected borders by Suzuki’s algorithm and (c) the rightmost window shows a mapped output for clarity.

From here, each of the borders, or blobs, which are designated within the system memory, are assigned to polygons using the Ramer-Douglas-Peucker algorithm to reduce the number of curves where the moment of each elements are recorded. Fig 3 shows an initialization exercise using the Graphical User Interface (GUI) to calibrate the algorithm with *Ae*. *aegypti* mosquitoes released into the chamber:

In this calibration exercise, however, some mosquitoes have not been detected due to the resolution limitation. This aspect of the quantification will be discussed in the following sections.

### C. Initializing and usage of software

**Fig 4:**
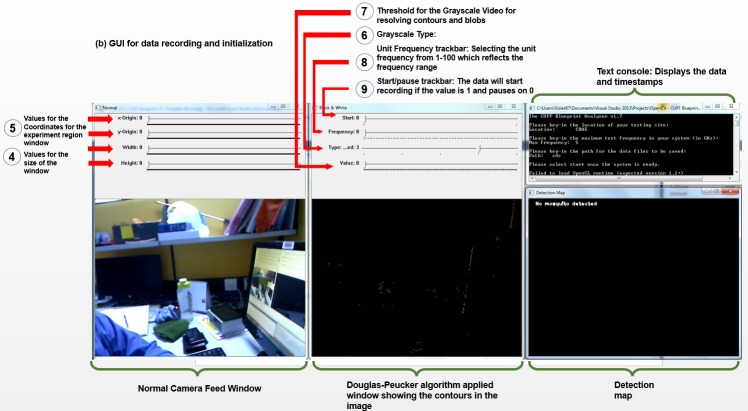
GUI of the application. (a) The input console for data input, (b) the GUI for initialization and recording the data.

Upon launching the software as mentioned in **Section B**, four windows are produced, including a normal camera feed window, a grayscale window where the filters are adjusted for the blobs to be detected from each frame, a mapping window and a text console. The GUI appears as shown in Fig 4:

Referring to Fig 4(A), upon starting the application, the console will require input of the (1) location, (2) frequency range (for indexing purposes of any test variables) and (3) the directory of the data files to be saved. After keying in the details, the GUI will appear as shown in Fig 4(B). The application is initialized by selecting the region which is of interest (4), which is selecting the chamber region which contains the mosquitoes. This is performed by rendering an empty boxed shape on the window, which is demonstrated in Fig 3(A) and 3(C), where the scanning region of the chamber are defined. However, there may be mosquitoes which reside near the sealed parts i.e foam or corners where the region is difficult to be detected, or some may be dormant. In this case, the mosquitoes which fall beyond the region defined within the software will be exempted for data simplicity. The box is then repositioned (5), where adjusting the *x*- and *y*-origin coordinates and the size of the region is performed back and forth until a satisfactory boundary is defined on the chamber. This also involved moving the camera around until a satisfactory distance and camera configuration is achieved.

From here, the grayscale image type is selected (Type 0: Binary, 1: Binary Inverted, 2: Truncate, 3: To Zero, 4: To Zero Inverted) which corresponds to preferences of the user. Usually Type 0, which is a binary form is selected. The contours from the image feed are shown on the window as highlighted in number (6). This is followed by selecting the threshold of the grayscale image (7), where the contours will show. Here, it is up to the user’s discretion to determine the accuracy of the threshold by comparing the detected blobs on the detection map and the normal video window. Normally some noise will be observed therefore will be subject to the user’s discretion.

After the configuration is complete, the data will start recording after sliding the track (9) from 0 to 1. This action also immediately creates a folder and a.txt file which saves the keyed-in information with the timestamps, each of the blobs’ coordinates and the average *x*- and *y*-coordinates as well. From here the text file is further processed for data visualization into spectral analysis as will be discussed in Section III.

### D. Mosquito preparation, release and stimuli

Before each testing sessions, approximately 200 specimens of female *Ae*. *aegypti* (Linnaeus) mosquitoes between 5–9 days of age where at this cycle known to be actively searching for a blood meal [33], were obtained from Parasitology Department, University of Malaya. The Bora-bora strain of *Ae*. *aegypti* which originated from Hawaii previously obtained from Universiti Sains Malaysia (USM) were used in this study. The *Ae*. *aegypti* were colonized under standard insectary conditions with 27 ± 2°C, 75–90% relative humidity and a 12 h: 12 h (light:dark) light cycles. The larvae were provided with a fine mixture of mice chow, beef liver, cat biscuit and milk powder in the ratio of 2:1:1:1 by weight, while the adults were provided with 10% sucrose solution. The mosquitoes are transferred into smaller containers and transported to the chamber lab.

The mosquitoes are recollected from the container using a glass aspirator with rubber tubing with gentle inhalation. The mosquitoes are trapped inside the glass tube with a copper mesh and exhaled into the chamber through a release port as shown in Fig 1. A plastic stopper is applied immediately after all the mosquitoes have been released into the chamber. This procedure is done for both the test and control regions, totaling approximately 100 specimens in both. After the mosquitoes are released, the chamber is closed and the mosquitoes are left to settle down in the environment for an hour.

For stimuli during tests, a human subject is placed near the release port of the intervention region of the chamber instead of odorized materials [6] for a more direct approach as per recommendation in testing mosquito response with the presence of a human as attractant [12]. Two sessions of data collection was carried out, the first with constant stimuli of human presence by positioning AA near the intervention region approximately 80 cm away from the intervention chamber, and the second session with periodic presence of AHP with approximately the same distance. The human subject makes periodic openings on the stopper for increased odor entry for both constant and periodic appearances. Each of the present session is recorded and added into the dataset for analysis. In this case, no ethics committee was required with the presence of human participants AA and AHP since complete isolation between the participants and the mosquitoes was established except for odor entry *per se*.

For the first session for continuous presence of stimuli, following the release of the mosquitoes in both chamber regions, the chamber and cameras were positioned for the system initialization as mentioned in Section B. A person AA is stationed beside the intervention chamber as shown in Fig 1 throughout the period for maintaining presence of human odor/heat, as was also performed in a previous study albeit odorized materials and non-human heat was generated for the experiment [2] After the thresholding is established, the data is recorded for approximately 40 minutes. The data output records the timestamps of ±2.00 second intervals. Although the data also records each of the blobs’ coordinates as shown in Fig 2, which may include the noise within the system, only the average distances of the blobs are visualized for an overall spectral view of the behavior pattern of the *Ae*. *aegypti*. In the second session, after the release of the mosquitoes in both regions and initializing sessions, AHP is stationed beside the intervention chamber periodically while introducing odor and heat into the chamber release port. Each proximity session lasts for a few recorded minutes, and the chamber is deserted for another 4–5 minutes. The rest of the procedures are in accordance with the continuous test. Both testing session lasted for 45 minutes.

## III. Results & discussion

### A. Constant presence of human odor versus mosquitoes’ positions

**Fig 5:**
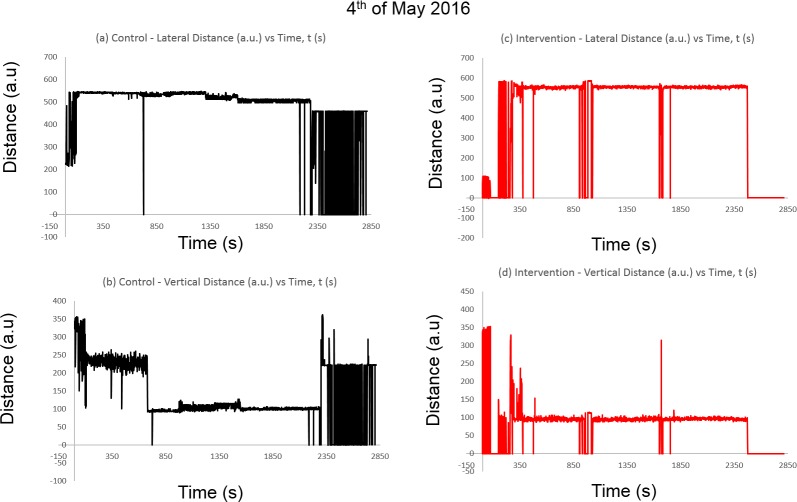
**The average distance (a.u) of the detected blobs against time (s) in:** (a) Lateral distance in Control Chamber, (b) Vertical distance in Control Chamber, (c) Lateral distance in the intervention chamber and (d) Vertical distance in the intervention chamber.

Fig 5 shows the spectral distribution of the collective distance in arbitrary units (a.u) as defined within the coordinate position of the raster scan versus time in seconds. See **S1 Dataset** for reference.

From the results, virtually constant readings can be observed. However, the constant readings from Fig 5(A) and 5(C) demonstrates one of the challenges of establishing a constant accuracy. This is due to the camera occlusion and lighting drift which produces the noisy output toward the end of the observation. In Fig 5(C), there are areas where the no output is observed due to the mosquitoes resting on the edges of the chamber, showing inactivity. In Fig 5(B), the vertical component shows more noise components than the effects of mosquito activity, whereas Fig 5(D) shows more erratic behavior among the mosquitoes initially, suggesting vertical movements are more abundant among the mosquitoes with the presence of human odor and heat, and the relatively constant readings which followed subsided, probably due to the constancy of the air and motionless stimuli. However, the relevance of this data depiction is maintained despite the effect of algorithmic noise. This is because similar to the noise in spectral analysis, a net mosquito behavior was obtained when it enters into the frame of observation, although this may be countered using a higher resolution system. A more detailed analysis will be covered in the discussion.

### B. Periodic presence of human odor versus mosquitoes’ positions

**Fig 6:**
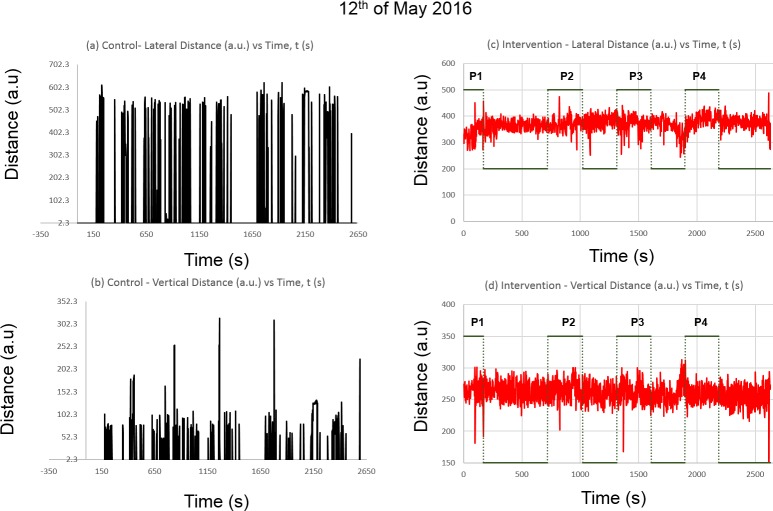
**The average distance (a.u) of the detected blobs in:** (a) Lateral distance in Control Chamber, (b) Vertical distance in Control Chamber, (c) Lateral distance and human presence (dotted line) in the intervention chamber and (d) Vertical distance and human presence (dotted line and designated as P1-P4) in the intervention chamber, all against time, t (s).

Fig 6 shows the spectra of the mosquito activity, with the indication of human presence.

Based on Fig 6(A) and 6(B), although the results from the control region seems different from the control in Fig 5, the constant peaks can be seen in Fig 6(A) whereas the gaps between the peaks indicates no detected blobs. This was due to the mosquito inactivity in the chamber. However, this was different in Fig 6(B), where there seems to some random distancing from the net blob detection, whereas the results indicate probable distant human detection due to the proximity of the regions. In Fig 6(C), there seems to be a complexity in the spectra during the presence of humans. However, as mosquito attraction is a very complex behavior, not all presence registers proximity in our observation. This is probably due to the delayed odor permittivity towards the mosquitoes, where the P1 indicates detection of human presence, while P2 registers attraction after the person has left the chamber, although P3 and P4 again registers attraction albeit in a nonlinear manner. However, the results yielded from Fig 6(C) and 6(D) indicates a clearer relationship on the periodic presence of human odor, as shown by the dotted line where a high dotted plateau (P1-P4) indicates the human presence whereas a low indicates non-presence. As also shown in Fig 5(D), this can be clearly seen from Fig 6(D) where three first appearances (P1, P2 and P3) shows more vertical flight activity, with more consistency. A more detailed analysis will be covered in the following section.

### C. Discussion

#### Setup issues and software development

The code development in this experiment was tailored for the purpose of mapping the average lateral and vertical position of the mosquitoes by using a single camera for each region of observation. This simplifies the technical complexities surrounding the use of multiple cameras for individual insect detection, which may have its advantages, but with many tradeoffs. Table 1 summarizes and compares several setup characteristics for tracking or quantification of insects’ motion, and finally compared with the development in this paper.

**Table 1:** Summary of the advantages and disadvantages of the methods used in previous literature in insects’ quantification of behavior.

Authors	Number of cameras for test area	Containing region size (WxLxH cm)	Software used	Main advantages	Main disadvantages for collective behavior analyses
Zou, D., Q. Zhao, et al. (2009) [22]	2	35x35x25	Custom Image Processing Software	• Improved 3D tracking on fruit flies using optimization methods	• Small area of observation • Source code not openly available
Dankert, H., L. Wang, et al. (2009) [27]	1	4x5x11.5	Not specified	• One camera only for tracking movements	• Small area of observation, inapplicable for mosquitoes.
Butail, S., N. Manoukis, et al. (2011 & 2012) [3, 24]	2	n/a (Open Area)	MATLAB	• Open source • Able to track many mosquitoes (n>3)	• Frequent Occlusions
Spitzen, J., C. W. Spoor, et al. (2013) [2]	2	160x60x60	‘Track3D’ (Noldus Information Technology, Wageningen, The Netherlands)	• Accurate 3D tracking	• Relatively complex setup • Limited number of mosquitoes which can be observed (n = 1)
Chambers, E., H. Bossin, et al. (2013) [5]	1	30×30× 30	ImageJ v. 1.43 software	• Open source software • Using only one camera	• Small area of observation
Wilkinson, D. A., C. Lebon, et al. (2014) [1]	1	30x40x10	MATLAB R2012b	• Open source • Using only one camera • Simpler to adapt for 2D mosquito flight tracking	• n/a
Cheng, X. E., Z.-M. Qian, et al. (2015) and Cheng, X. E., S. H. Wang, et al. (2015) [34, 35]	3	36x36x36	Core View™ online console software	• Highly accurate 3D tracking • Able to estimate flight orientation	• Requires more than 2 cameras • High data sizes
Khan, B., J. Gaburro, et al. (2015) [20]	2	Not specified	OpenCV	• Open source	• n/a (Not enough information)
Crall, J. D., N. Gravish, et al. (2015) [26]	1	21.5 x 15.0 (height not specified)	MATLAB	• Open source • Highly detailed flight profiles and orientation identification • Low-cost	• Specific for bees which have pattern signatures on the bodies, inapplicable for smaller insects • Small area of observation
Parker, J. E., N. Angarita-Jaimes, et al. (2015) [4]	1	Not specified, performed in a room	StreamPix software (www.norpix.com)	• Using only one camera • Able to classify several types of motion eg. Swooping, bouncing, resting, visiting	• Complex camera setup • Specialized software
Angarita-Jaimes, N., J. Parker, et al. (2016) [19]	1	200x100x140	MATLAB,StreamPix software	• Open source • Using only one camera • Able to classify mosquito behavioral modes in a large area	• High data size (1 TB/hour of video data) • Requires specialized camera setup
Method in this paper, core algorithm by Suzuki, S. (1985)	1	176x47x18	OpenCV 3.0 and Visual Studio 2013	• Low-cost • One camera per region of observation • Open source • Straightforward and simple setup • Small data file size output (<5 MB/hour)	• Occlusion and noise problems

Referring to Table 1, several studies used specialized software for such experiments, such as the ‘Track3D’ (Noldus Information Technology, Wageningen, The Netherlands) [2], ‘Lucia G’ image processing software by Nikon for studying larvae of *Anopheles stephensi* mosquitoes [36] or Streampix software used to study *Anopheles gambiae* and *Culex quinquefasciatus* mosquitoes on bed nets [4, 19]. The main disadvantage with these software are inherent to the specificity of the setup which results in many unnecessary restrictions in the experiment, Also, the software are usually cost-prohibitive and may require high-performance computers for the operation. Issues with data size arise when high resolution images are rendered from high-end equipment, which takes form in multiple *jpeg* images [34] and other video file formats [2] Several open source developments for mapping insect positions are also available, which focused on identifying certain insect species such as bees [26] and fruit flies [27, 34] using pattern recognition algorithms, in contrast to the development used in this study The development in this paper, however, can be used for other insect species as well, since the heuristic used in this setup is guided by the size of the perceived blobs within the raster scan, although more conditions may be incorporated for higher accuracy. However, mapping mosquitoes within the chamber poses several challenges including the resolution and occlusion issues due to the small size of these specimens, unlike imaging bees, which has a better chance at being tracked with unique signatures on the body, combined with the relatively larger size of the specimens compared to mosquitoes which eases the detection algorithm [26]. This was also the concern raised in other studies regarding diffuse imaging induced by image occlusion [24]. Also, similitude in insect appearance has been one of the most severe challenges in establishing the veracity of the findings, as highlighted in a study on 3D tracking of fruit flies [34, 35], and this reflects the difficulty in tracking mosquitoes which is relatively much smaller in size. Capturing pixels of the mosquitoes within the image scan has high noise levels, as also highlighted in a study on 3D flight reconstruction on mosquito swarms [24]. Another study on *Anopheles gambiae* and *Culex quinquefasciatus* mosquitoes’ attacking behavior mentioned occlusion problems with surrounding objects, such as bed nets and the suspension string surrounding the region of observation, which contributes to the missing positions of mosquitoes in several instances [19]. Several researchers attempted on mitigating severe occlusion problems in 3D tracking of animal groups by devising a novel Global and Recursive Tracking Algorithm (GReTA), where possible links between multiple frames are computed for obtaining the path of the individual in the group [21]. Several steps can be made in order to improve the data accuracy. Several studies obtained more detail from the test subjects by placing camera in close proximity towards the region of observation [5, 27, 34]. In a study on 3D mosquito tracking, camera placement was also concluded as one of the most important factors in obtaining accurate mosquito positions [20].This includes adjusting the distance of the camera towards the chamber to increase the captured pixels of the mosquitoes captured by the imaging process. However, the disadvantages of this method includes the potential loss of data, although mosquitoes falling out from the vision is considered as inactive as declared in some studies [2]. Potential loss of data, which may result from a 2D observation, also can be mitigated by limiting the length of the chamber, as performed by Wilkinson et al. (2014) by setting the length to only 10 cm, whereas in this study was set to 18 cm [1]. Another method to ensure data accuracy is to use function-specific cameras, such as the use of monochrome cameras [2, 4, 34], high-resolution types with multiple angles [22, 24, 26, 34], or even the use of a single high resolution camera [1] which may be not a viable option for entomologists who are opting for low-cost solutions. However, multiple camera setups are more appropriate for individual insect tracking, which may not be necessary for aggregation purposes, in this case for collective behavior. Refer to Table 1 for the summary on the cameras used in the setup. In our case, the balance between adjusting threshold and distance has to be manually performed until a satisfactory mapping is established. For adaptors of this software development, a smaller chamber may be desirable, as designed by several researchers [5, 27, 34], which ultimately optimizes the pixels captured from the camera for a higher accuracy in the data collection. However, this may compromise the data representation due to the lack of space which is needed for investigating spatial response of the mosquitoes, as some exhibit more erratic behavior only after getting within 30cm of human odor, as shown with *Anopheles gambiae* mosquitoes [2]. As summarized in Table 1, all included previous research which housed mosquitoes have at least 30 cm of chamber width.

### Comparison of control regions in both continuous and periodic presence of stimuli

For the controls in the continuous mode in Fig 5(A), there is collectively erratic lateral movement of approximately 300 units (a.u) observed from the data during the first four minutes, which suggests the initial activity of the mosquitoes due to the constant presence of stimuli. This was followed by a nearly constant reading until a non-presence was observed during the 12^th^ minute, which suggests that most of the mosquitoes are out of the frame of observation, and possibly accustomed towards the constant surrounding. This was shortly followed by a constant reading again until 37 minutes of the test, which then showed another erratic behavior until the end of the experiment, ranging approximately 500 units of fluctuating distances. Fig 5(B), however, which represents vertical mosquito positions, exhibits longer erratic behavior up to 11 minutes. However, similar to the lateral motions, the mosquitoes appeared to show stagnancy until the 37^th^ minute. This was followed by a similar erratic behavior until the experiment ceased. In Fig 6(A), however, the lateral movements show more intermittent and erratic behavior. A constant peak is always observed with intervals of 40–50 seconds, suggesting periodic blurring or occlusion in the image detection. However, this also suggests the lack of radical changes in the mosquitoes’ lateral positions.

For periodic presence of stimuli as shown in Fig 6(B), however, shows less erratic behavior in the vertical flights, where during the first 3 minutes, only up to 100 units of vertical displacement was observed, which increased to 200 units during the 7^th^ minute, and 250 units during the 12^th^ minute, and peaking to around 300 units during the 20^th^ minute, which again can be observed during the 30^th^ minute. This was followed by a slight dip up to 150 units during the 36^th^ minute, and another sharp increase near the end of the experiment. The intermittent activity observed indicates that vertical movements are more significant in determining the response of mosquitoes, as shown in studies on flight paths of mosquitoes against human odor [2]. In comparison, a study on *Ae*. *polynesiensis* mosquitoes’ landing target preference suggests that mosquitoes less prefer landing on white targets compared to targets colored in red, navy blue or black, which reports a landing time between 1,7–8.3 minutes per landing on white-colored control targets [5], which is consistent with the data observed for vertical motions in the white-colored chamber background used in this study, where resting behavior can be deducted with the absence of any reading, with a resting period of approximately 3–8 minutes.

In conclusion, comparing the controls in both tests in Fig 5(A) and 5(B) and Fig 6(A) and 5(B), although the results from the control regions in both instances seems to be completely random, the constant peaks in Fig 6(B) indicate the similar static or inactivity of the mosquitoes which can be seen in Fig 6(A) whereas the gaps between the peaks indicates no detected blobs. In Fig 5(A) and 5(B), the motions detected by the system is more continuous, meaning it registers virtually no net displacement of the swarm behavior. The random gaps in the data in Fig 6(B) indicates that no mosquitoes are residing within the range of view of the borders defined from the matrix, where most mosquitoes have tendencies to rest on the edges of the chamber. Previous studies also emphasized that mosquitoes spend most their time resting [1], as also indicated on the near-constancy on most parts of the data, as observed the controls for both continuous and periodic presence of stimuli. This corresponds quite well with the 3D tracking of *Anopheles gambiae* mosquitoes which reports on relatively noncomplex and random flight paths of mosquitoes without any odor or heat introduced into the chamber [2]. A study which analyzes the flight path of mosquitoes on insecticide-treated bed-nets also affirms relatively low activity in bed-nets without any human bait, indexed in terms of swooping, bouncing, resting and visiting, although swooping rates are similar between bed-nets with and without human bait [4]. In our case, however, the noise which is present as indicated in Fig 6(A) where occasional activity of the mosquitoes are recorded and in effect disrupted the spectrum. However, the potential of this method is maintained due to noise cancellation algorithms which may be applied in this spectra.

### Comparison of intervention region in both continuous and periodic presence of stimuli

For the intervention region in the continuous mode in Fig 5(C), the aggregate lateral position of the mosquitoes show similar initial sporadic behavior until approximately 6 minutes fluctuating around 500 units (a.u), followed by a stagnancy until the 15^th^ minute. This was followed by abrupt null and continued its course of near constant position until around 30 minutes into the experiment, again followed by the abrupt apparent non-presence of mosquitoes in the frame. This was followed by another identical constancy until around 43 minutes of the experiment, followed by another indication of non-presence in the region of observation until the end of the experiment, which suggests the mosquitoes’ shifting into the edges of the chamber. This effect was also reported in a study on baited bed nets against mosquitoes, where a constant presence of a human has a higher percentage of *Anopheles gambiae* mosquitoes only visiting or being stationary on the bed net [4] which probably indicates the tendency for mosquitoes to acclimatize and acquiesce with constant stimuli presence. As for the lateral positions in periodic tests shown in Fig 6(C), where the presence of the stimuli is indicated by the green line with binary states indicating presence, an initial stimuli presence (designated P1) showed a movement away from the source around 100 units, before returning to the mean of the spectrum, which may seem counterintuitive towards the attractant effect of human odor. However, this effect was attested in previous studies which demonstrate the complex behavior of the mosquitoes in reaction towards human odor and heat, where lateral and vertical flight speeds reportedly increase albeit in a convoluted path, but no linear attractant effect was observed [2, 4]. This was followed by a relatively insignificant fluctuations in the movement until another similar peak was observed during the 11^th^-15^th^ minute where the region of presence P2 is indicated, again during presence of stimuli with another shift towards the stimuli as indicated by the following peak. Another sharp dip in the spectrum, indicating movement towards the stimuli position was also observed albeit without the presence of the stimuli itself, falling slightly out of P2. This was probably due to the lingering human odor and heat after leaving the experiment site. This was followed by a similar shift, although less significant with a range of around 100 units during the 21^st^-26^th^ minute as indicated by P3, and another shift towards the source during P4 (31^st^-36^th^ minute), although occurring only during the beginning of the presence of stimuli.

For vertical flights in the intervention region in the continuous mode as shown in Fig 5(D), fluctuations in the vertical flights can be observed from the start until 6 minutes. This is followed by a near-constant reading until 15 minutes into the experiment, followed by apparent non-presence for a brief moment and resuming another constant collective resting behavior until the 28^th^ minute, where a sharp peak indicating a sharp upward movement around 350 units. This was followed by another constant reading at roughly 100 units until the 41^st^ minute. This was followed by similar constant reading for the lateral positions with Fig 5(C) at 43 minutes, and another indication of non-presence until the end of the experiment. For the periodic tests as shown in Fig 6(D), a significant shift of around 100 units in the vertical movement was registered during P1 which occurred until 6 minutes into the experiment. This was followed by an expected insignificant fluctuation ranging between 0–50 units of the spectrum until P2 which occurred during 11^th^-16^th^ minute, registering a slightly lesser-valued downward motion of around 70 units during the presence of stimuli. Again, another relatively constant behavior can be seen without the stimuli until P3 occurring during 21^st^-26^th^ minute as indicated by P3, with a downward shift of about 150 units, the biggest shift in the spectrum observed. After another period of stagnancy until P4, occurring during the 31^st^-36^th^ minute, another slight downward movement was observed, occurring during the start of presence of stimuli. This was followed by stagnancy until the end of the experiment at 45 minutes. In this instance, a more significant effect of stimuli is observed with vertical flights as reported previously, especially when the mosquitoes reach within 15 cm from the stimuli [2]. However, in this experiment, the presence of the stimuli is 80 cm away from the chamber, suggesting a higher correlation between stimuli presence and the increase in the vertical flight activity was due to the use of an actual human subject, which introduces carbon dioxide (CO_2_) into the experiment environment. This is supported by increased *Anopheles gambiae* and *Ae*. *aegypti* mosquitoes’ landing rates with the presence of human-odorized materials and CO_2_ [18, 37].

In conclusion, comparing the intervention parts in both cases in Fig 5(C), Fig 5(D) and Fig 6(C) and Fig 6(D), there is a significant relation between the presence of odor and heat with the overall collective motion spectra, especially in Fig 6(C) and 6(D), which highlights the feasibility to adopt this method for collective mosquito behavior, although patterns of the intervention in Fig 5(C) and 5(D) are less erratic, most probably due to the constant stimuli presence, where mosquitoes have been shown to acclimatize towards the surroundings if human odor is present [18]. This is indicated by the pattern of the spectrum which shifted towards the source of odor as shown in Fig 6(D) on the left side of the chamber (Fig 1) during the presence of a human, and the suggestive increase in vertical flights as shown in Fig 6(A). In addition, although attraction by human odor is shown to be positive, the reaction of the mosquitoes take time between consecutive presences made. Again, the flight activity of the mosquitoes have shown to concur with the test on flight paths of *Anopheles gambiae* mosquitoes which exhibit more vertical flight patterns as compared to other components in the presence of human odor and heat [2], as also depicted in the flight pattern of the same species against baited bed-nets, which show more tortuous flights [4]. Comparing the collective behavior in 2D as shown in Fig 6(C) and 6(D), this demonstrates the quantitative attractant property of human odor, heat and CO_2_. This was also asserted by a study on automated flight path analysis of *Ae*. *aegypti* mosquitoes against CO_2_ plumes, where the attraction of thermal bodies is strongly dependent on the presence of CO_2_ [18, 37].

## IV. Conclusions and future strategies

In conclusion, this study has demonstrated the potential advantages of using a collective behaviour analysis by employing Suzuki’s method of topological structure extraction analysis by border following, a function which is openly available in OpenCV^TM^ software [30]. By testing this development on the effect of human presence as a stimuli against the mosquitoes’ activity, the findings show that the mosquitoes’ vertical flights increase in presence of humans, particularly if periodic appearances were made, which corroborates with a previous study on *Anopheles gambiae* malaria mosquitoes [2]Furthermore, the findings in this study also substantiates another study that the use of human subjects returns a higher correlative effect in showing the attractant effect of CO_2_ as a stimuli against mosquitoes’ vertical flight activity [18, 37]. The quantification exercise only requires two standard-issue USB-connected web cameras to a personal computer (PC) which is able to operate a basic version of Visual Studio^TM^ 2013 and OpenCV^TM^ software, of which both can be used for free indefinitely. The chamber design in this case is built with low-cost materials, with minimal expertise required on assembling the setup.

A fully functional and lightweight source code is provided for entomologists to adopt and improve upon for collective insect behavior analysis. The data files produced from this system requires minimal disk space (typically less than 5 MB-sized text file for a 2-hour data collection). Although false positives varies in our system which affects a fully accurate analysis, a spectral pattern can still be observed with this method, where further pattern recognition algorithms and artificial intelligence may be used to classify the collective behavior.

Based on our findings, mosquitoes’ collective behavior prove to be easily quantifiable using 2D imaging rather than using a much more complex 3D analysis, where a spectral analysis of the average moment of the mosquito swarm can be used to derive many forms of behavior, potentially for attraction or repellency tendencies as well. Disclosure of the source code (See **S1 Source Code and Files**) is provided for potential improvements by adaptors of this method.

## Supporting information

S1 Source Code and Files This code is written in C++ in Visual Studio Community 2013 environment with OpenCV^TM^ 3.0, although both versions are updated from time to time and the software can be used for newer versions as well. Both these software are open source and can be used indefinitely where free versions are available online for download. Several tutorials for initializing the software for direct use of the code are setup online as well for potential adaptors to this method. The code is supplied with the Visual Studio 2013 project files for direct access. The files are provided in a WinRAR compressed format (.rar).
(RAR)

S1 Dataset This data is exported into an Excel workbook (.xlsx) for maneuverability. The data for both testing sessions (4^th^ and 12^th^ of May 2016) are included.
(XLSX)
